# Adverse renal outcomes following targeted therapies in renal cell carcinoma: a systematic review and meta-analysis

**DOI:** 10.3389/fphar.2024.1409022

**Published:** 2024-06-26

**Authors:** Song Ren, Xiuling Chen, Yang Zheng, Tingwei Chen, Xu Hu, Yunlin Feng, Shangqing Ren

**Affiliations:** ^1^ Department of Nephrology and Institute of Nephrology, Sichuan Academy of Medical Sciences and Sichuan Provincial People’s Hospital, School of Medicine, University of Electronic Science and Technology of China, Sichuan Clinical Research Centre for Kidney Diseases, Chengdu, China; ^2^ Robotic Minimally Invasive Surgery Center, Sichuan Provincial People’s Hospital, University of Electronic Science and Technology of China, Chengdu, China; ^3^ Department of Urology, Institute of Urology, West China Hospital, Sichuan University, Chengdu, China

**Keywords:** renal dysfunction, proteinuria, targeted therapy, renal cell carcinoma, systematic review, meta-analysis

## Abstract

**Introduction:** To clarify the prevalence of adverse renal outcomes following targeted therapies in renal cell carcinoma (RCC).

**Methods:** A systematic search was performed in MEDLINE, EMBASE, and Cochrane Central Library. Studies that had reported adverse renal outcomes following targeted therapies in RCC were eligible. Outcomes included adverse renal outcomes defined as either renal dysfunction as evidenced by elevated serum creatinine levels or the diagnosis of acute kidney injury, or proteinuria as indicated by abnormal urine findings. The risk of bias was assessed according to Cochrane handbook guidelines. Publication bias was assessed using Funnel plot analysis and Egger Test.

**Results:** The occurrences of the examined outcomes, along with their corresponding 95% confidence intervals (CIs), were combined using a random-effects model. In all, 23 studies including 10 RCTs and 13 observational cohort studies were included. The pooled incidence of renal dysfunction and proteinuria following targeted therapies in RCC were 17% (95% CI: 12%–22%; I^2^ = 88.5%, *p* < 0.01) and 29% (95% CI: 21%–38%; I^2^ = 93.2%, *p* < 0.01), respectively. The pooled incidence of both types of adverse events varied substantially across different regimens. Occurrence is more often in polytherapy compared to monotherapy. The majority of adverse events were rated as CTCAE grades 1 or 2 events. Four studies were assessed as having low risk of bias.

**Conclusion:** Adverse renal outcomes reflected by renal dysfunction and proteinuria following targeted therapies in RCC are not uncommon and are more often observed in polytherapy compared to monotherapy. The majority of the adverse events were of mild severity.

**Systematic Review Registration:** Identifier CRD42023441979.

## Introduction

Renal cell carcinoma (RCC) ranks as the sixth most frequently diagnosed cancer in men and the tenth in women worldwide (Siegel et al., 2018). The incidence has been increasing, with up to 17% of patients had distant metastasis at the time of diagnosis (Capitanio and Montorsi, 2016; Capitanio et al., 2019). Notably, significant progress has been made in the treatment of RCC, particularly metastatic RCC, over the past decade, primarily through the development of targeted therapies based on biological pathway research (Capitanio and Montorsi, 2016). Targeted therapies have emerged as the current mainstays of care, demonstrating efficacy in achieving durable complete responses (Capitanio and Montorsi, 2016).

Despite of the substantial efficacy, targeted therapies in RCC are associated with various adverse events (AEs), among which fatigue, hypertension, gastrointestinal discomfort, dysphonia, and palmar-plantar erythrodysaesthesia are the most commonly reported (Ruiz et al., 2014; Krawczyk et al., 2023). In contrast to the high incidence in approximately one-third of the population, AEs in patients receiving targeted therapy for RCC are not given enough attention. A national survey in oncologists in the United Sates reported a although it is customary for oncologists to discuss adverse events with patients, less than half of the physicians proactively initiate these discussions (Ruiz et al., 2014).

Adverse renal outcomes are also observed following targeted treatment in RCC patients, including impaired renal function and proteinuria. Persistent presence of adverse renal events might lead to the discontinuation of targeted therapies. Therefore, understanding the overview of renal AEs following targeted therapies is helpful not only for consultation on clinical decision-making prior to the initiation of targeted therapies, but also for subsequent patient management. However, there is a lack of summary on the evidence regarding the frequency of adverse renal outcomes following targeted therapies in RCC in literature.

In light of this background, we undertook this comprehensive review and meta-analysis to clarify the incidence of unfavorable renal outcomes following targeted therapies in RCC in trial settings. Our objective was to enhance understanding of this subject matter and furnish substantiated evidence for clinical practice.

## Materials and methods

### Data sources and searches

A comprehensive search was undertaken to identify relevant studies published until July 13^th^, 2023 in MEDLINE via PubMed, EMBASE via Ovid, and Cochrane Central Library via Ovid, adhering to the guidelines outlined in the Preferred Reporting Items for Systematic Review and Meta-Analyses (PRISMA) statement (Liberati et al., 2009). The search utilized appropriate text terms related to the names and targeted molecules of commercially available pharmaceuticals for targeted therapies and renal cell carcinoma (see Supplementary Table S1). No restrictions were imposed on publication date or language. The search was prospectively registered on PROSPERO and amended (Identifier# CRD42023441979).

### Study selection

This systematic review considered studies that had reported targeted therapies in the context of renal cell carcinoma and adverse renal outcome following treatment as eligible. Adverse renal outcomes included renal dysfunction reflected by increased serum creatinine or definitions of renal failure and proteinuria. Both observational cohort studies and randomized controlled trials (RCTs) were included, without any restrictions on study population, type of targeted therapy, or targeted molecules of treatment.

The screening process was conducted independently by two reviewers (S.R. and S.Q.R.) using a standardized approach. The titles and abstracts of all retrieved records from the database search were meticulously examined. Exclusions were made for duplicates, pediatric studies, non-original studies (such as reviews, editorials, commentaries, guidelines, proceedings, and secondary analysis of published trials), case reports, study protocols, conference abstracts lacking sufficient information, *in vitro* studies, animal studies, studies unrelated to cancer, and cancer studies that had not reported kidney injury outcomes or any targeted therapy. Additionally, the reference lists of articles reviewed in their entirety were manually scrutinized to identify any relevant studies. Any discrepancy was adjudicated by a third reviewer (Y.L.F.).

### Outcome

The outcome in this systematic review was adverse renal outcomes following targeted therapies, defined as either renal dysfunction as evidenced by elevated serum creatinine levels or the diagnosis of acute kidney injury (AKI), acute renal failure (ARF), or renal failure, or proteinuria as indicated by abnormal urine findings. These outcomes were quantified using their incidences reported in each study cohort.

### Data extraction and quality assessment

Two independent reviewers (S.R. and S.Q.R.) extracted data from eligible studies and compiled them into a shared document. Any discrepancy was resolved by the third reviewer (Y.L.F.).

The collected data included various elements such as the authors’ names, publication year, geographical location, total number of patients in the study population, specifics of the targeted therapies employed, and the occurrence of adverse renal outcomes following treatments. In the case of RCTs, the extracted data also encompassed the number of patients in both interventional and control groups, details regarding interventional and control treatments, and incidences of adverse renal outcomes observed within each respective group. Additional information regarding potential sources of heterogeneity, such as the demographic composition and average age of the study population, was also gathered for the purpose of conducting sensitivity analysis.

### Critical appraisal

Two reviewers (S.R. and S.Q.R.) independently assessed the risk of bias of included studies using the Agency for Healthcare Research and Quality (AHRQ) tool (Chou et al., 2010). Any discrepancy was resolved by consensus.

### Data synthesis and analysis

Data analysis and synthesis were performed using Stata (version 14.0) and Review Manager (RevMan 5.2) software. Since we aimed to identify the pooled incidences of the outcomes, each study group in the RCTs was treated as an independent cohort, from which the incidences of adverse renal outcomes were collectively meta-analyzed along with those observed in the observational cohort studies. The occurrences of the examined outcomes, along with their corresponding 95% confidence intervals (CIs), were combined using a random-effects model. Additionally, subgroup analyses were performed based on the severity of adverse events evaluated by the Common Terminology Criteria for Adverse Events (CTCAE) and classification of targeting agents. Due to substantial variations in targeted therapies across RCTs, it was not feasible to directly compare the study outcomes among different treatment regimens. Consequently, the targeted treatments were categorized based on their respective target molecules. The degree of statistical heterogeneity was assessed using the I^2^ statistic (Ioannidis, 2008). The pooled incidences of the study outcomes were classified as having low, moderate, and high statistical heterogeneity based on I^2^ values of <25%, between 26% and 75%, and >75%, respectively (Ioannidis, 2008). Publication bias was assessed using Funnel plot analysis and Egger Test. A two-sided *p*-value of < 0.05 was considered statistically significant.

## Results

### Search findings

**FIGURE 1:**
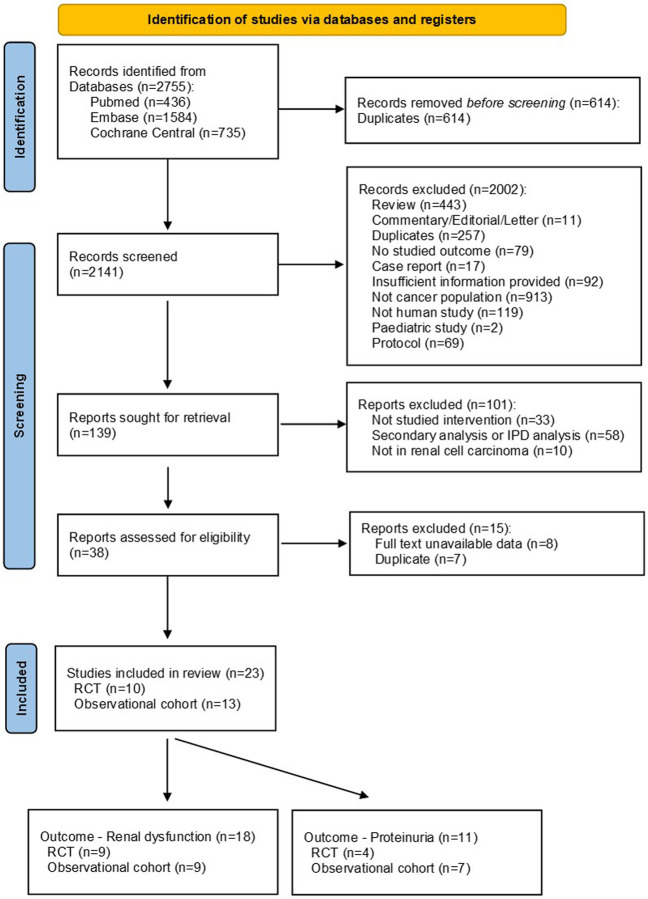
PRISMA flow chart for study selection. Abbreviations: IPD, individual patient data; RCT, randomized controlled trial.

A total of 2,141 records were initially identified through literature searching and after removing duplicates. 2002 records were excluded after screening the titles and abstracts, and another 115 publications were further excluded following full text review. Finally, 23 studies were incorporated into this systematic review and meta-analysis, including 10 RCT studies (Motzer et al., 2014; Motzer et al., 2015; Choueiri et al., 2016; Powles et al., 2016; Jonasch et al., 2017; Bergmann et al., 2020; Choueiri et al., 2022; Lee et al., 2022; Pal et al., 2022; Sheng et al., 2023) and 13 observational cohort studies (Hainsworth et al., 2010; Harzstark et al., 2011; Ryan et al., 2011; Molina et al., 2012; Guo et al., 2013; Hainsworth et al., 2013; Harshman et al., 2013; Molina et al., 2014; Motzer et al., 2016; Oudard et al., 2016; Oyama et al., 2017; Hutson et al., 2021; Pedersen et al., 2021) (see Figure 1).

### Study characteristics

Five studies and 12 studies had only provided data on proteinuria (Hainsworth et al., 2010; Harshman et al., 2013; Molina et al., 2014; Motzer et al., 2015; Motzer et al., 2016) and renal dysfunction (Ryan et al., 2011; Molina et al., 2012; Guo et al., 2013; Motzer et al., 2014; Oudard et al., 2016; Powles et al., 2016; Jonasch et al., 2017; Oyama et al., 2017; Bergmann et al., 2020; Pedersen et al., 2021; Choueiri et al., 2022; Lee et al., 2022), respectively. Six studies had reported both outcomes (Harzstark et al., 2011; Hainsworth et al., 2013; Choueiri et al., 2016; Hutson et al., 2021; Pal et al., 2022; Sheng et al., 2023). The targeted therapies were classified into seven groups based on the targets of regimens, including mTOR inhibitors, Tyrosine kinase inhibitor (TKI), a combination of mTOR inhibitors and TKI, AKT inhibitors, and a combination of mTOR inhibitors with either PI3K inhibitors, GLS1 inhibitors and VEGF/HER2 inhibitors, among which the first three groups were the mainstays. Detailed characteristics of the included studies are shown in (Table 1).

**TABLE 1:** General characteristics of studies included in meta-analysis.

Study	Country	Study design	Population	Targeted agents	Population	Gender (M/F)	Age	Kidney related outcomes	ROB
Bergmann et al. (2020)	Germany	RCT	Non-clear cell renal cell carcinoma	Temsirolimus	12	8/4	59.5	CTCAE grade 3 Renal events	Low
				Sunitinib	10	8/2	65.5		
Choueiri et al. (2016)	United States of America	RCT	Advanced renal cell carcinoma	Cabozantinib	331	253/77	63	Blood creatinine increased, proteinuria	High
				Everolimus	322	241/86	62		
Choueiri et al. (2022)	United States of America	RCT	Advanced clear cell renal cell carcinoma	Everolimus	32	26/40	66	Acute kidney injury	Unclear
				Sapanisertib	32	22/39	61		
				Sapanisertib + TAK-117	32	25/41	66		
Guo et al. (2013)	China	Cohort	Renal cell carcinoma	Everolimus	64	44/20	52	Increased blood creatinine	Low
Hainsworth et al. (2013)	United States of America	Cohort	Advanced clear cell renal carcinoma	Pazopanib	55	42/13	60	Renal failure, proteinuria	Unclear
Hainsworth et al. (2010)	United States of America	Cohort	Advanced renal cell carcinoma	Bevacizumab + Everolimus	80	60/20	64	Proteinuria	Unclear
Harshman et al. (2013)	United States of America	Cohort	Refractory metastatic renal cell carcinoma	Bevacizumab + Everolimus	10	9/1	55	Proteinuria	Unclear
Harzstark et al. (2011)	United States of America	Cohort	Metastatic clear cell renal cell carcinoma	Everolimus + Sorafenib	20	15/5	65	Creatinine increased, proteinuria	Unclear
Hutson et al. (2021)	United States of America	Cohort	Non-clear cell renal cell carcinoma	Lenvatinib + Everolimus	31	20/11	64	Creatinine increased, proteinuria	Unclear
Jonasch et al. (2017)	United States of America	RCT	Renal cell carcinoma	MK-2206	29	21/8	59	Creatinine increased	High
				Everolimus	14	14/0	63.5		
Lee et al. (2022)	United States of America	RCT	Renal cell carcinoma	Telaglenastat + Everolimus	46	37/9	65	Blood creatinine increased	High
				Placebo + Everolimus	23	20/3	65		
Molina et al. (2012)	Canada	Cohort	Renal cell carcinoma	Sunitinib + Everolimus	20	16/4	62	Creatinine elevation	High
Molina et al. (2014)	Canada	Cohort	Metastatic renal cell carcinoma	Lenvatinib (E7080) + Everolimus	20	14/6	58	Proteinuria	Low
Motzer et al. (2016)	United States of America	Cohort	Metastatic renal cell carcinoma	Everolimus + Sunitinib	58	43/15	58	Proteinuria	High
Motzer et al. (2014)	United States of America	RCT	Metastatic renal cell carcinoma	Everolimus followed by sunitinib	238	166/72	62	Increased blood creatinine	Low
				Sunitinib followed by everolimus	233	176/57	62		
Motzer et al. (2015)	United States of America	RCT	Metastatic renal cell carcinoma	Lenvatinib + Everolimus	51	35/16	61	Proteinuria	High
				Lenvatinib	52	39/13	64		
				Everolimus	50	38/12	59		
Oudard et al. (2016)	France	Cohort	Metastatic renal cell carcinoma	VEGFR-TKI + Everolimus	165	116/49	65	Renal Failure	High
Oyama et al. (2017)	Japan	Cohort	Advanced renal cell carcinoma	Everolimus	53	34/19	64	Creatinine increased	High
Pal et al. (2022)	United States of America	RCT	advanced renal cell carcinoma	14 mg Lenvatinib + Everolimus	172	133/39	61	Blood creatinine increased, proteinuria	Unclear
				18 mg Lenvatinib + Everolimus	171	129/42	62		
Pedersen et al. (2021)	United States of America	Cohort	Renal cell carcinoma	Everolimus + Vorolanib	22	11/11	57	Elevated creatinine	High
Powles et al. (2016)	United Kingdom	RCT	Metastatic renal cell carcinoma	AZD2014	26	22/4	58	Blood creatinine increased	Low
				Everolimus	23	19/4	63		
Ryan et al. (2011)	United States of America	Cohort	Advanced renal carcinoma	Everolimus + Imatinib	19	16/3	65	Elevated creatinine	Unclear
Sheng et al. (2023)	China	RCT	Metastatic renal cell carcinoma	Vorolanib + Everolimus	133	102/31	58	Elevated creatinine, proteinuria	Low
				Vorolanib	133	107/26	59		
				Everolimus	133	103/30	59		

Abbreviations: F, female; M, male; RCT, randomized controlled trial; ROB, risk of bias.

### Renal dysfunction following targeted therapies

**FIGURE 2:**
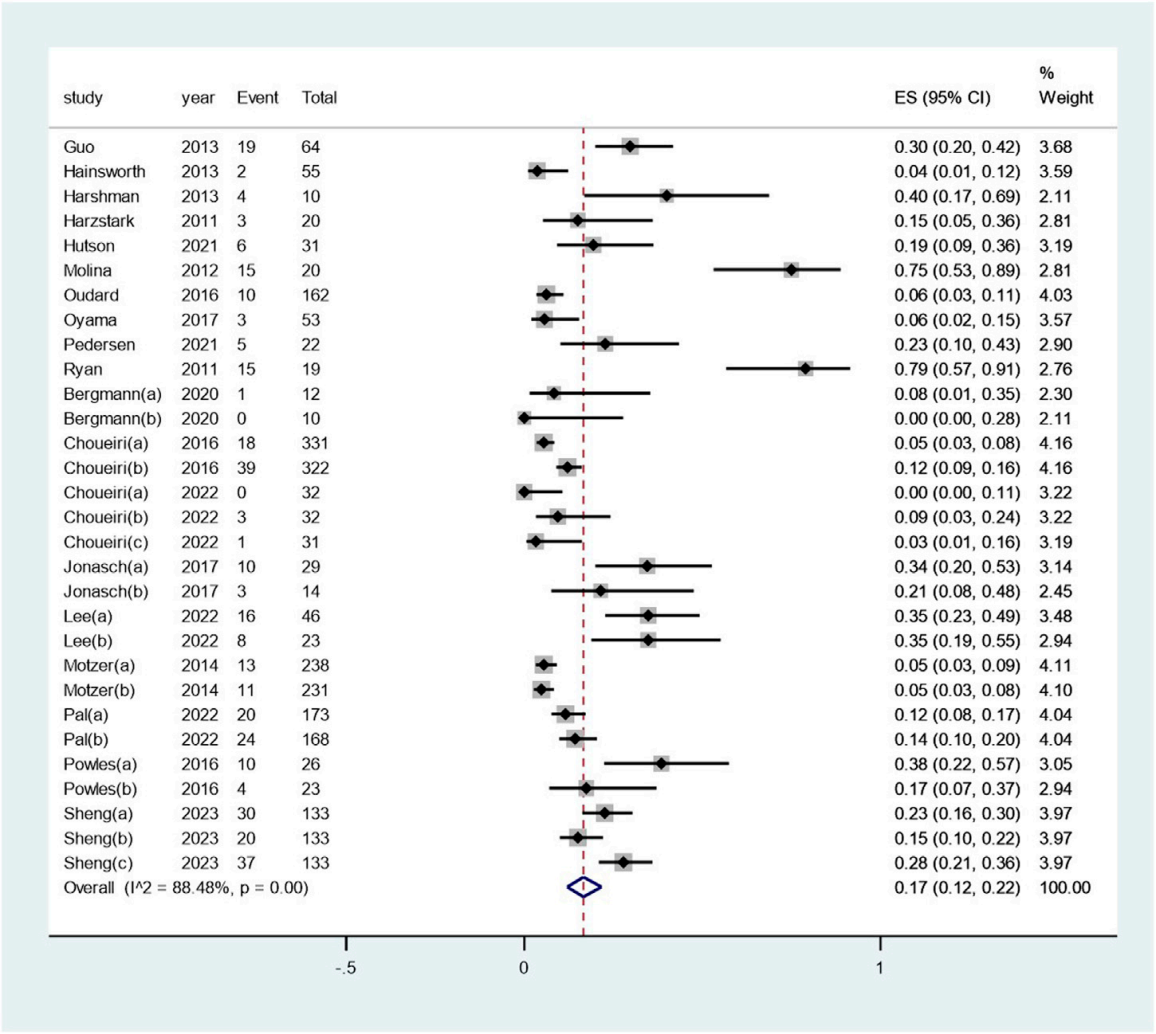
Aggregated occurrence rate of renal dysfunction subsequent to the administration of targeted therapies in renal cell carcinoma. Notes: The pooled incidence of renal dysfunction following targeted therapies was 17% (95% CI: 12%, 22%), with a high degree of heterogeneity observed among the studies (I^2^ = 88.5%, *p* < 0.01).

**FIGURE 3:**
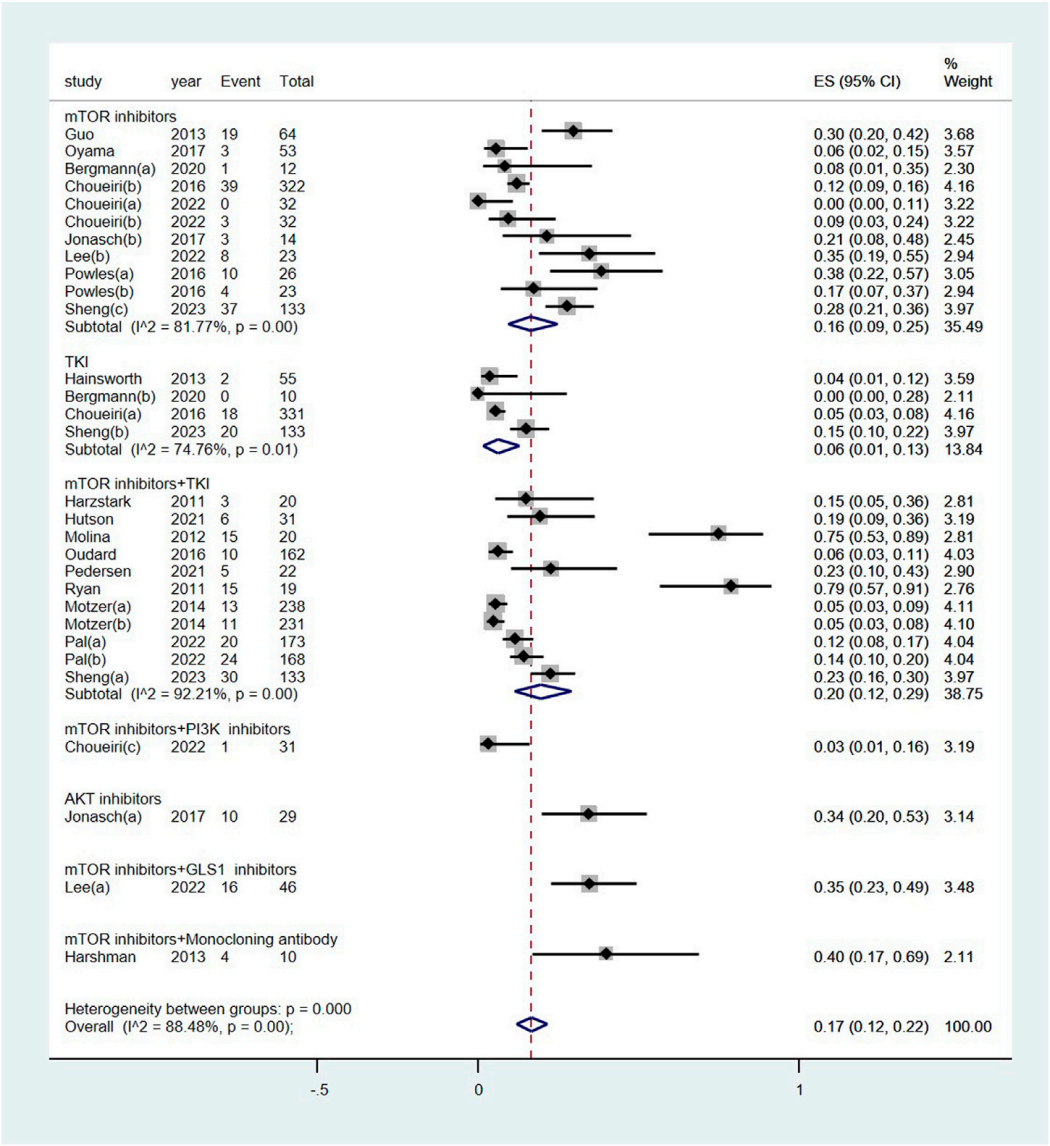
Subgroup examination of renal dysfunction following targeted therapies in renal cell carcinoma, categorized according to the classification of targeted agents. Notes: The targeted therapies are classified into seven subgroups based on their targets.

The pooled incidence of renal dysfunction following targeted therapies was 17% (95% CI: 12%, 22%), with a high degree of heterogeneity observed among the studies (I^2^ = 88.5%, *p* < 0.01) (see Figure 2). Notably, the incidences of renal dysfunction varied substantially across different regimens of targeted therapy, ranging from 0.03% to 40% (heterogeneity between sub-groups: *p* < 0.01) (see Figure 3). Further analysis comparing the incidences of renal dysfunction among three regimens that had been reported in more than three studies revealed a range of 6%–20% (see Supplementary Figure S1). The pooled incidences of renal dysfunction events, categorized as CTCAE grade 1–2 and grade 3–4, were determined to be 15% (95% CI: 10%, 20%) and <1% (95% CI: 0, 1%), respectively (see Supplementary Figure S2).

### Proteinuria following targeted therapies

**FIGURE 4:**
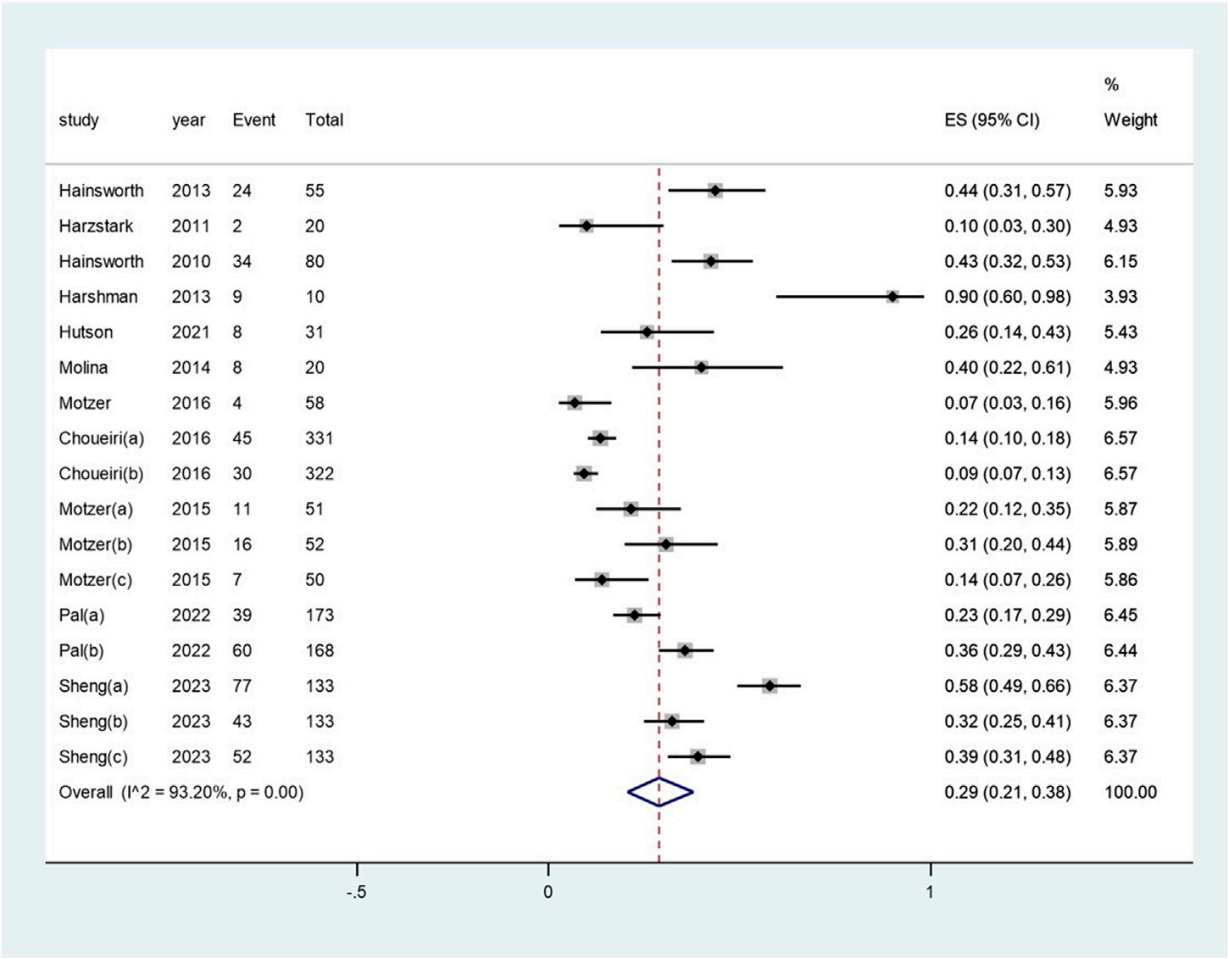
Aggregated occurrence rate of proteinuria subsequent to the administration of targeted therapies in renal cell carcinoma. Notes: The pooled incidence of proteinuria was 28% (95% CI: 2o%, 36%), with a high degree of heterogeneity observed among the studies (I^2^ = 92.9%, *p* < 0.01).

**FIGURE 5:**
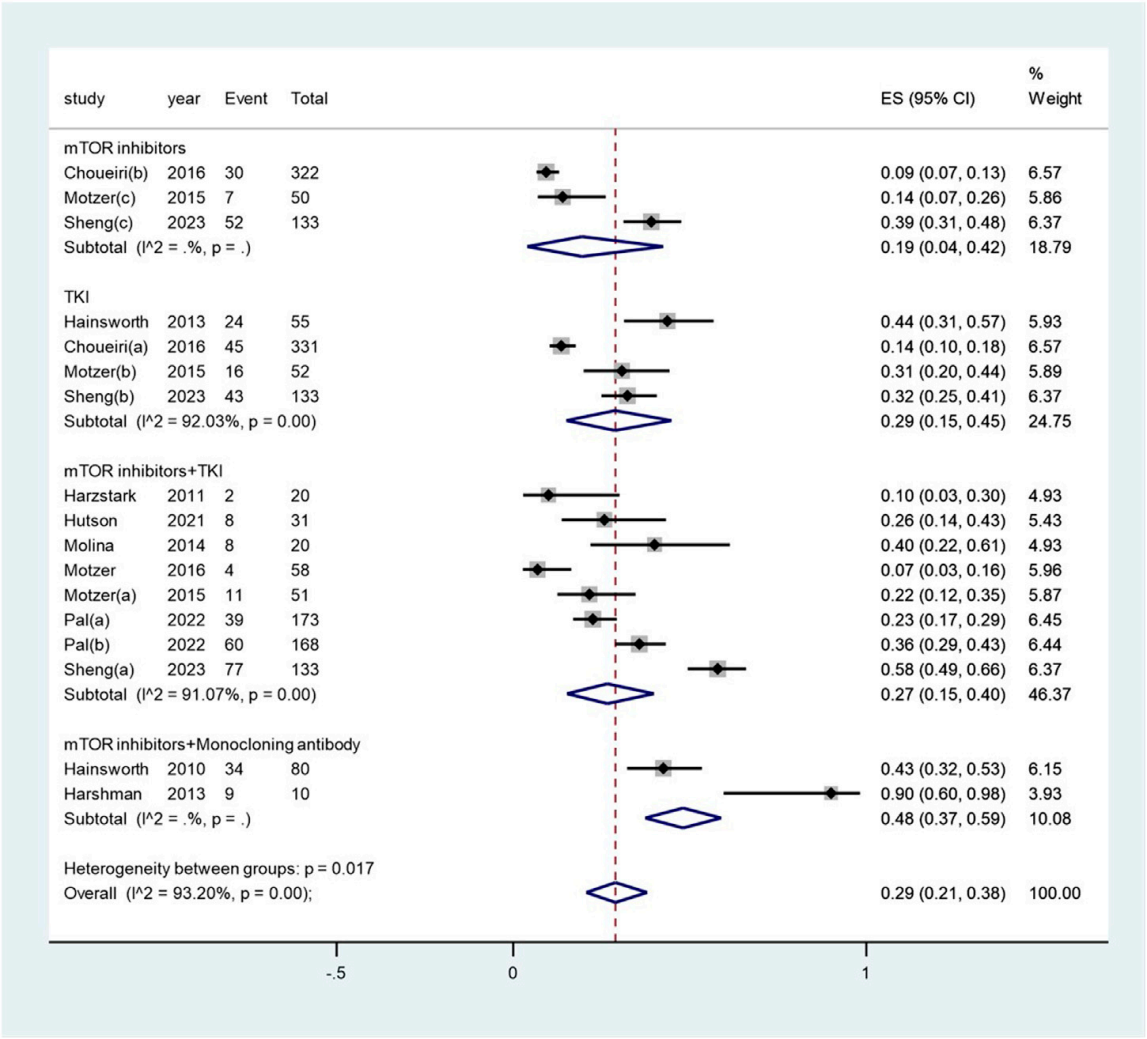
Subgroup examination of proteinuria following targeted therapies in renal cell carcinoma, categorized according to the classification of targeted agents.

The pooled incidence of proteinuria subsequent to the administration of targeted therapy was 29% (95% CI: 21%, 38%), with a high degree of heterogeneity observed among the studies (I^2^ = 93.2%, *p* < 0.01) (see Figure 4). The pooled incidence of proteinuria following targeted therapy substantially ranged from 19% (95% CI: 4%, 42%) after mTOR inhibitor treatment to 48% (95% CI: 37%, 59%) after a combination of mTOR inhibitor and monoclonal antibody treatment (heterogeneity between sub-groups: *p* = 0.017) (see Figure 5). The pooled incidences of proteinuria events, categorized as CTCAE grade 1–2 and grade 3–4, were deemed to be 21% (95% CI: 15%, 28%) and 7% (95% CI:3%, 11%), respectively (see Supplementary Figure S3).

### Risk of bias assessment

The evaluation based on the AHRQ tool identified six, eight, and nine studies with low, unclear, and high risk of bias, respectively (Supplementary Figure S4).

### Publication bias

The funnel plot revealed symmetry on visual inspection, suggesting the absence of publication bias (Supplementary Figure S5). This observation was supported by the results of the Egger test (*p* = 0.10, Supplementary Figure S6).

## Discussion

Our findings suggested adverse renal outcomes are rarely reported in targeted therapy in RCC, not being a focused topic in clinical trials or pharmacovigilance; however, the incidence is not low. The pooled occurrence rates of renal dysfunction measured by elevated serum creatinine or diagnosis of AKI or ARF and proteinuria were up to 17% and 29%, respectively. Renal dysfunction was more commonly observed in the mTOR inhibitors and TKIs polytherapy compared to either monotherapy, whereas proteinuria occurred at a similar rate in the combination of mTOR inhibitors and TKI compared to the TKI monotherapy. The majority of the adverse events were CTCAE grades 1 or 2 events. Nearly one-fourth of the included studies in this meta-analysis were rated as having low risk of bias.

Although kidney related adverse events are not the leading adverse events in studies on targeted therapy in RCC (Di Lorenzo et al., 2011), the findings of this study and previous studies consistently suggest these events are not uncommon (Ruiz et al., 2014; Krawczyk et al., 2023; Semenescu et al., 2023). The reported rate of proteinuria is higher than renal function impairment. The occurrence of impaired renal function might be underestimated due to the fact that AKI defined using the creatinine-based criteria is often left undiagnosed, particularly when the increase of creatinine achieves the diagnostic criteria (i.e., 26.5 μmol/L) yet the highest level of creatinine is still below the upper limit. Pharmacovigilance studies on check-point inhibitors (CIs), which belong to another important class of therapies for RCCs, have reported high prevalence of AKI following CI treatments (Hu et al., 2021; Zhu et al., 2022), which is also more commonly seen in polytherapy (Hu et al., 2021). Therefore, adverse renal outcomes are worthy noted in applying targeted therapies in RCC patients and should be closely monitored.

Despite of the relatively high occurrence, the severity of renal adverse events in this clinical setting is not alerting. Our findings showed most of the adverse events are grades 1 or 2, not necessitating drug treatment. This is consistent with our observations in clinical practice, in which the elevated serum creatinine level following targeted therapies rarely exceed 150 μmol/L in most patients with previously normal renal function. Although the elevation of creatinine exhibits a clear relationship with the timing of targeted therapies, the treatment is mainly close monitoring and supportive regimens, such as sufficient uptake of fluid and controlling risk factors for kidney injury (Ruiz et al., 2014). In most cases, the creatinine stops to increase, sometimes gradually decreases, even to normal range. Renal replacement therapy is extremely rare. The results of this meta-analysis provide evidence for this management strategy of close follow up of targeted therapies in the RCC population.

The occurrence of adverse renal outcomes subsequent to targeted therapies in RCC results from a combination of various factors. The underlying reasons are multifaceted in pathophysiological and clinical perspectives. Firstly, targeted agents, such as sorafenib and sunitinib in the included trials in this meta-analysis have been reported to cause thrombotic microangiopathy (Garcia and Atallah, 2017; Genest et al., 2023), which is an important cause of proteinuria and acute decline in renal function. Secondly, the gastrointestinal discomfort including diarrhea and vomiting can cause dehydration, reducing the kidney perfusion, thus increasing the risk of pre-renal kidney injury (Di Lorenzo et al., 2011). Thirdly, the toxicity of targeted agents is also an important source of injury in this setting. Renal biopsy studies indicated AKI in CIs treatment is most commonly induced by acute tubulointerstitial nephritis, either alone or accompanied by other renal lesions including acute tubular injury or glomerular lesions (Cortazar et al., 2016; Moss and Perazella, 2022). However, there is a lack of renal biopsy study on pathological manifestation of kidney injury in targeted therapies in RCC, most likely because these adverse events were not severe enough for conducting invasive kidney biopsy. With the advances of pharmacological research, the underlying mechanisms of targeted agents for kidney injury might also evolve (Moss and Perazella, 2022). Fourthly, common risk factors for kidney injury, for example, the diabetes, hypertension, senior age, and the use of NSAIDs, proton pump inhibitors, and ACEi/ARBs are frequently observed in cancer patients and can all increase the risk of renal dysfunction (Chen et al., 2023). The exploration of potential reasons also reminds us the importance to manage risk factors when prescribe targeted therapies in RCC patients, take necessary measures to alleviate the hazard effects of damaging factors, and pay attention to close monitoring.

To our best acknowledgment, this is the first systematic review and meta-analysis on the occurrence of adverse renal outcomes following targeted therapies in RCC. Our results benefited from a comprehensive literature search and unbiased comparisons in RCTs. There are still some limitations worth mentioning. First, the results were limited by the reporting in the included studies. Although the funnel plot analysis did not suggest the existence of publication bias, as mentioned earlier, since mild elevation in serum creatinine and proteinuria might be asymptomatic thus being left undiagnosed, the reported incidence might have been underestimated. Second, there are variations on definitions of renal dysfunction and proteinuria across the studies. Some studies even just simply reported the adverse renal events as *increased creatinine* or *proteinuria* without details in the definitions. The variations might have been an important source of the observed high heterogeneity and must be considered in the interpretation of the results. Third, the proportion of included studies with low risk of bias was only one-fourth in this meta-analysis, precluding from making robust conclusions. Future studies covering renal adverse outcomes following targeted therapies in RCC will help us gain more insight into the appropriate clinical management approaches.

## Conclusion

In summary, the results indicate that adverse renal outcomes including renal dysfunction and proteinuria are not infrequent in RCC patients receiving targeted therapies, particularly in cases of polytherapy as opposed to monotherapy. The majority of these adverse events were of mild severity. The results remind us to take appropriate measures to mitigate risk factors for renal injury and closely monitor the outcome of adverse events in this population and are awaiting confirmation with real-world clinical data.
