# Supplementary material for: Adverse renal outcomes following targeted therapies in renal cell carcinoma: a systematic review and meta-analysis
Source: Front Pharmacol. 2024 Jun 26;15:1409022. doi: 10.3389/fphar.2024.1409022 (PMC11234087; doi:10.3389/fphar.2024.1409022)

**Supplementary Materials**

eTable 1. Search terms used in Embase via Ovid.

eFigure 1. Pooled incidences of renal dysfunction among three regimens that had been reported in more than three studies.

eFigure 2. Pooled incidence of renal dysfunction based on CTCAE grades following targeted treatment.

eFigure 3. Pooled incidence of proteinuria based on CTCAE grades following targeted treatment.

eFigure 4. Risk of bias assessment of included studies using the AHRQ tool.

eFigure 5. Funnel plot for kidney injury estimates in this meta-analysis.

eFigure 6. Egger test for kidney injury estimates in this meta-analysis.

eTable 1. Search terms used in Embase via Ovid.

| 1 | "*tinib".mp. |
| --- | --- |
| 2 | "*umab".mp. |
| 3 | "*fenib".mp. |
| 4 | "*imab".mp. |
| 5 | "*ciclib".mp. |
| 6 | everolimus.mp. |
| 7 | "*parib".mp. |
| 8 | "*lisib".mp. |
| 9 | "*cept".mp. |
| 10 | abiraterone.mp. |
| 11 | "*omab".mp. |
| 12 | "*ostat".mp. |
| 13 | "*degib".mp. |
| 14 | miraparib.mp. |
| 15 | 1 OR 2 OR 3 OR 4 OR 5 OR 6 OR 7 OR 8 OR 9 OR 10 OR 11 OR 12 OR 13 OR 14 |
| 16 | EGFR.mp. |
| 17 | HER1.mp. |
| 18 | ERBB1.mp. |
| 19 | HER2.mp. |
| 20 | ALK.mp. |
| 21 | MET.mp. |
| 22 | ROS1.mp. |
| 23 | BRAF.mp. |
| 24 | MEK.mp. |
| 25 | VEGFR.mp. |
| 26 | VEGF.mp. |
| 27 | ERBB4.mp. |
| 28 | RET.mp. |
| 29 | CDK.mp. |
| 30 | mTOR.mp. |
| 31 | PARP.mp. |
| 32 | PI3K.mp. |
| 33 | TROP-2.mp. |
| 34 | KIT.mp. |
| 35 | PDGFR.mp. |
| 36 | RAF.mp. |
| 37 | PIGF.mp. |
| 38 | ABL.mp. |
| 39 | FLT.mp. |
| 40 | GFR.mp. |
| 41 | FGFR.mp. |
| 42 | Nectin-4.mp. |
| 43 | CYP17.mp. |
| 44 | CSF1R.mp. |
| 45 | GD-2.mp. |
| 46 | Smoothened.mp. |
| 47 | PTCH.mp. |
| 48 | RANKL.mp. |
| 49 | ROS1.mp. |
| 50 | TRK.mp. |
| 51 | 16 OR 17 OR 18 OR 19 OR 20 OR 21 OR 22 OR 23 OR 24 OR 25 OR 26 OR 27 OR 28 OR 29 OR 30 OR 31 OR 32 OR 33 OR 34 OR 35 OR 36 OR 37 OR 38 OR 39 OR 40 OR 41 OR 42 OR 43 OR 44 OR 45 OR 46 OR 47 OR 48 OR 49 OR 50 |
| 52 | "Randomized Controlled Trial*".mp. |
| 53 | "random*".mp. |
| 54 | "Phase III".mp. |
| 55 | "Phase 3".mp. |
| 56 | 52 OR 53 OR 54 OR 55 |
| 57 | "acute kidney injury".mp. |
| 58 | "acute renal failure".mp. |
| 59 | renal.mp. |
| 60 | kidney.mp. |
| 61 | "nephrop*".mp. |
| 62 | 57 OR 58 OR 59 OR 60 OR 61 |
| 63 | 15 AND 51 AND 56 AND 62 |

Note: This strategy will be adapted to identify trials in other electronic databases.

eFigure 1. Pooled incidences of renal dysfunction among three regimens that had been reported in more than three studies.

eFigure 2. Pooled incidence of renal dysfunction based on CTCAE grades following targeted treatment.


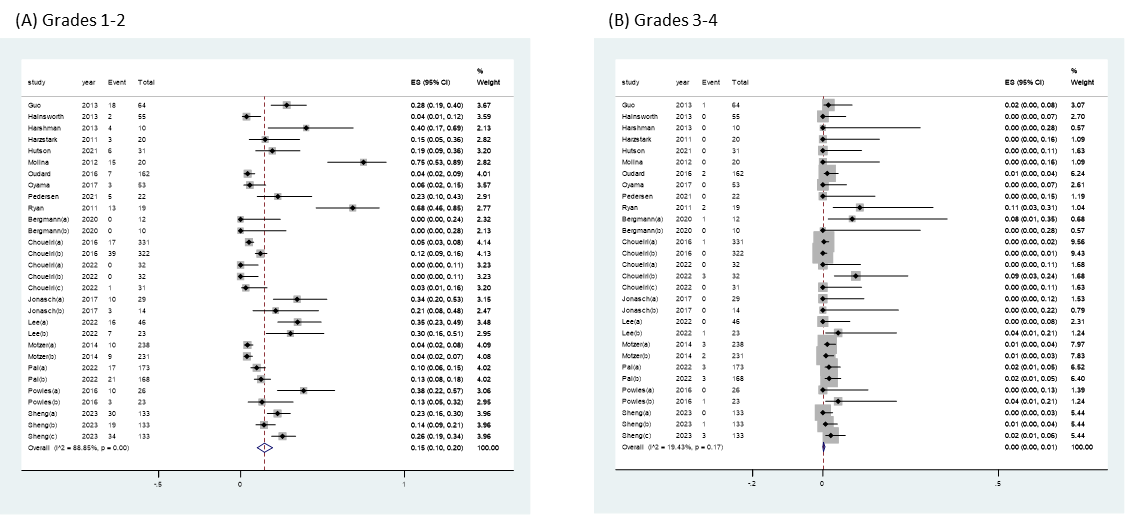


eFigure 3. Pooled incidence of proteinuria based on CTCAE grades following targeted treatment.


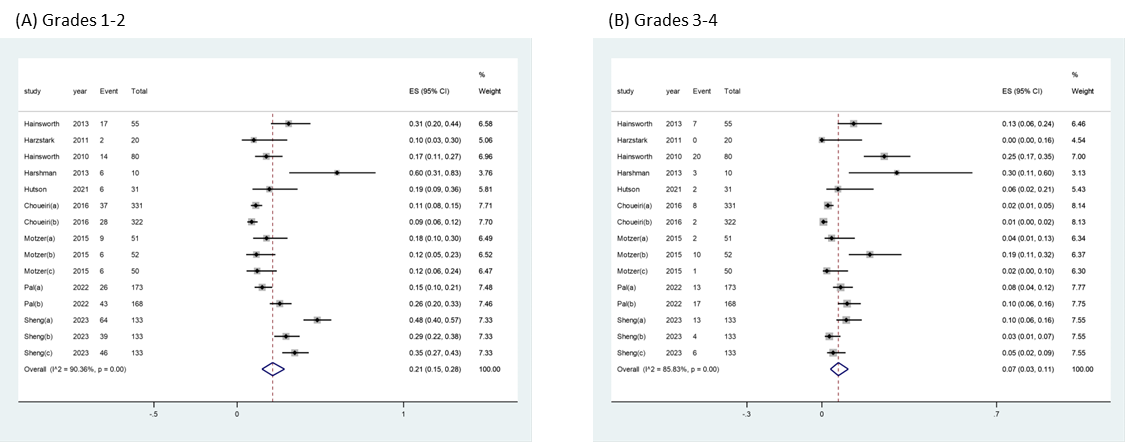


eFigure 4. Risk of bias assessment of included studies using the AHRQ tool.


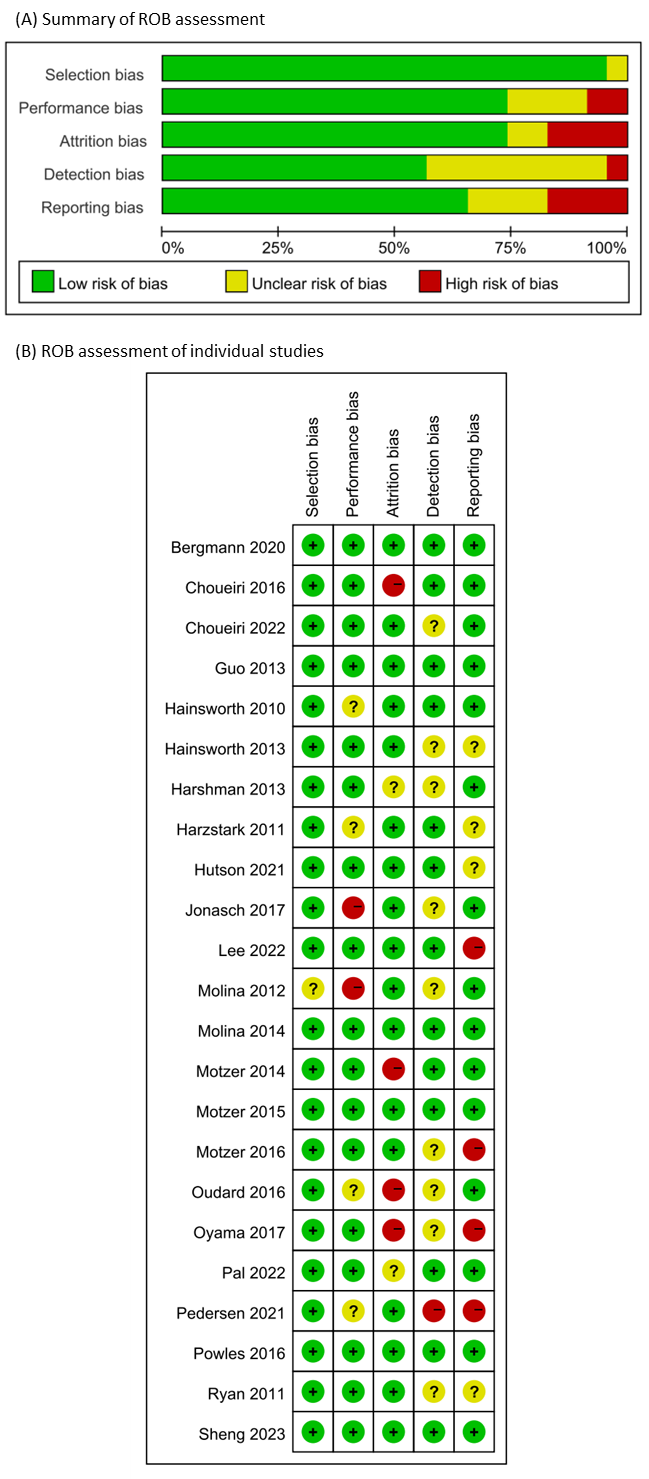


Note: (A) Summary graph of the ROB analysis; (B) ROB assessment of individual study.

eFigure 5. Funnel plot for kidney injury estimates in this meta-analysis.


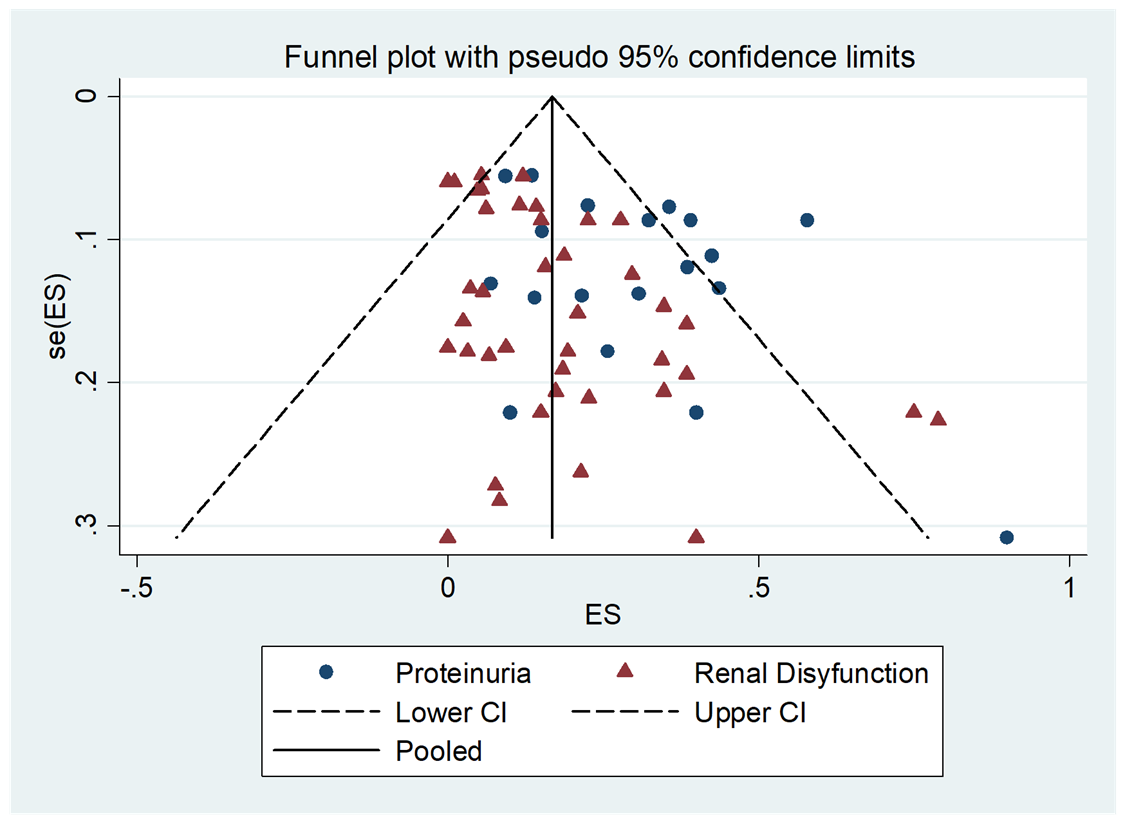


eFigure 6. Egger test for kidney injury estimates in this meta-analysis.


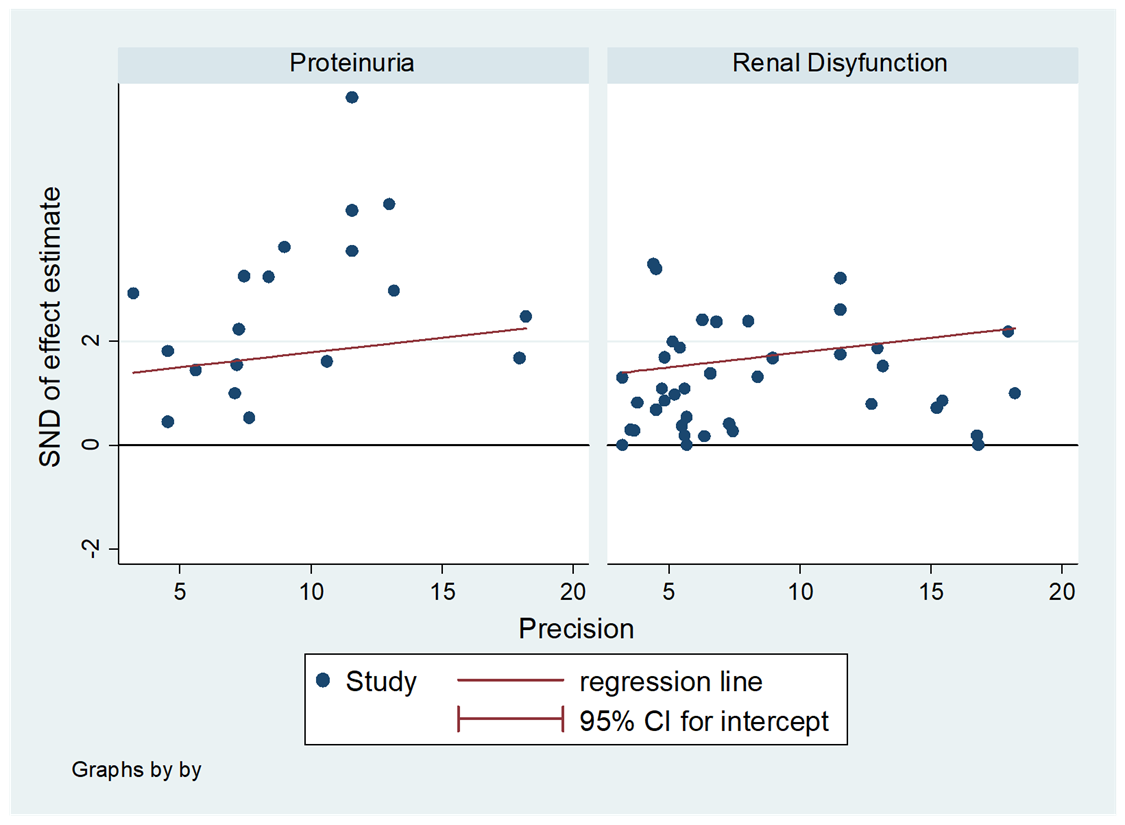

Supplement: Supplementary file 1 [file DataSheet1.docx]
